# Medical Aid in Dying: Europe’s Urgent Medico-Ethical Challenge

**DOI:** 10.3389/ijph.2023.1606538

**Published:** 2023-09-05

**Authors:** Uwe Güth, Shaun McMillan, Edouard Battegay

**Affiliations:** ^1^ Faculty of Medicine, University of Basel, Basel, Switzerland; ^2^ Department of Breast Surgery, Brust-Zentrum Zürich, Zurich, Switzerland; ^3^ International Center for Multimorbidity and Complexity in Medicine (ICMC), University of Zurich, Zurich, Switzerland; ^4^ Department of Psychosomatic Medicine, University Hospital Basel, Basel, Switzerland; ^5^ Merian Iselin Klinik Basel, Basel, Switzerland

**Keywords:** medical aid in dying, assisted suicide, euthanasia, physician assisted suicide, ethical issues

Medical aid in dying (MAID) or assisted dying is defined as the circumstance in which a physician prescribes or provides a lethal substance to a patient to enable the patient to end his or her life. The critical point about assisted suicide is that those wishing to die must themselves carry out the last, decisive life-ending act of the procedure. In contrast, voluntary active euthanasia also allows physicians or healthcare professionals to administer the lethal drug to the patient [1].

MAID is a comparatively new societal and medical phenomenon. More than almost any other medical-ethical topic, views and exchanges about assisted dying are intensely controversial [2, 3].

Proponents view its legalization as an achievement of a present-day society to emphasize an individual’s autonomy, also in making a conscious decision to end their life on their own terms. In contrast, opponents argue that MAID violates mainstays of medicine in which healing, managing pain and alleviating suffering, but never knowingly or intentionally causing harm or death, are non-negotiable cornerstones of medical practice.

Throughout the past two decades, most Western countries have experienced a steady increase in the acceptance of MAID for patients who suffer from a terminal disease and/or from irreversible, intolerable symptoms and functional limitations [4, 5]. Consequently, an increasing number of European countries, namely, Switzerland, Austria, Netherlands, Belgium, Luxembourg, and Spain allow for different forms of assisted dying under certain conditions [6–8]. Currently, based on the populations of these six countries, approximately 95 million people have legal access to an applicable form of MAID. In the coming years, more Western European and Scandinavian countries are likely to alter or adjust their laws to allow for specified forms of MAID, although their governments have not yet regulated assisted dying.

Observations of MAID in countries with corresponding long-term experience show that MAID has evolved from a rare marginal phenomenon to a societal mainstream. The number of persons who not only allow themselves to think about this formerly taboo option, but actually choose it, is growing rapidly and steadily. Recent data from the Netherlands shows that MAID accounts for nearly 5% of all deaths; in Switzerland this rate is 2% [9, 10].

Increasing longevity (associated with increased age-related multimorbidity), living outside a supportive family environment, or without a partner could result in MAID accounting for at least 5% of all deaths in the next 10–15 years in countries with legal access to MAID. However, these sociodemographic developments do not fully explain the expected rise for MAID cases to increase. The much more important influencing factor regarding attitudes towards MAID in the future is a shift in social values, in which religious convictions play a decreasing role, whereas personal freedom and autonomy to live one’s life become decisive life principles. This development has been observed since the 1960s and 1970s. The generation who is now entering their final stages of life has grown up in an environment where they were able to define their lives much more freely than their parents and grandparents. The latter were much more constrained in shaping their life and lifestyle through social class, with minimal opportunities for higher education, stringent gender roles, economic hardships, strong religious convictions and community pressures, among others.

Until recently, the possibility to “chart the course of one’s life” was reserved for a select few in the upper social class. This privilege now has become generally available. Parallel and consistent in this movement towards increased personal freedom for many, this autonomy then also includes the option to “chart the course of one’s life, including death.” Some individuals who have been accustomed to making their own decisions throughout their lives, may not allow former socially accepted standards to dictate what they might have to endure due to illness or old age-related complaints, and MAID will possibly no longer be seen as taboo but rather as a “natural option.”

Whatever one’s own position concerning MAID, we have to deal with it legally, emotionally, and as a society. In our opinion, it will be the most urgent medical-ethical challenge for the West in the coming years ahead, to guide and modulate MAID’s evolving design and development. With this guidance, healthcare providers (physicians, nurses, geriatric and palliative care providers, and other healthcare workers) will have to position themselves in terms of their own ethical framework and principles, and they will have to seek answers about how they deal with those who approach them with their wish to die. Of fundamental importance in this process are the convictions that society deems acceptable for legal MAID. The considerations for an individual’s request to receive MAID must be carefully weighed and ultimately limited to a certain degree in order to protect people from causing unnecessary harm to themselves and their families. Furthermore, the health profession must also ensure that those from its ranks who are directly involved in the complex and demanding practice of MAID are provided assistance through appropriate training, education, and support.

Where are we today in the international public health literature about MAID?

Due to the sociomedical originality of the phenomenon of MAID, there are many aspects in the global use of assisted dying which have not yet been sufficiently reviewed [11]. Therefore, now would be an important time to compile and evaluate long-term experiences of countries that have been providing their citizens with legal access to MAID. These empirical and evidence-based reports are fundamental for countries with legal access to MAID, as they monitor critical trends and developments that will only become more apparent as assisted dying becomes increasingly widely practiced. This information is equally valuable for countries that are now debating legalizing MAID in the near future, whereas these practical public health case examples can be incorporated into their future legislation and regulations.

## References

[1] Bosshard G Jermini D Eisenhart D Bar W. Assisted Suicide Bordering on Active Euthanasia. Int J Leg Med (2003) 117(2):106–8. 10.1007/s00414-002-0346-3 12690508

[2] Fontalis A Prousali E Kulkarni K. Euthanasia and Assisted Dying: What Is the Current Position and What Are the Key Arguments Informing the Debate? J R Soc Med (2018) 111(11):407–13. 10.1177/0141076818803452 30427291 PMC6243437

[3] Dugdale LS Lerner BH Callahan D. Pros and Cons of Physician Aid in Dying. Yale J Biol Med (2019) 92(4):747–50. 31866790 PMC6913818

[4] European Values Study. Justfiable: Euthanasia (2023). Available from: https://www.atlasofeuropeanvalues.eu/maptool.html (Accessed August 06, 2023).

[5] Cohen J Van Landeghem P Carpentier N Deliens L. Public Acceptance of Euthanasia in Europe: A Survey Study in 47 Countries. Int J Public Health (2014) 59(1):143–56. 10.1007/s00038-013-0461-6 23558505

[6] Mroz S Dierickx S Deliens L Cohen J Chambaere K. Assisted Dying Around the World: A Status Quaestionis. Ann Palliat Med (2021) 10(3):3540–53. 10.21037/apm-20-637 32921084

[7] Irish Hospice Foundation Paper. The International Experience of Assisted Dying (2021). Available from: https://hospicefoundation.ie/wp-content/uploads/2021/10/International-Experience-of-Assisted-Dying-Oct-2021.pdf (Accessed August 06, 2023).

[8] British Medical Association. Physician-Assisted Dying Legislation Around the World (2021). Available from: https://www.bma.org.uk/media/4402/bma-where-is-pad-permitted-internationally-aug-2021.pdf (Accessed August 06, 2023).

[9] Montagna G Junker C Elfgen C Schneeberger AR Güth U. Long-Term Development of Assisted Suicide in Switzerland: Analysis of a 20-Year Experience (1999-2018). Swiss Med Wkly (2023) 153:40010. 10.57187/smw.2023.40010 36971666

[10] Regional Euthanasia Review Committees. Annual Reports 2002-2021 (2022). Available from: https://english.euthanasiecommissie.nl/the-committees/documents/publications/annual-reports/2002/annual-reports/annual-reports (Accessed August 06, 2023).

[11] Güth U Junker C Schafroth M McMillan S Schneeberger AR Elfgen C ICD-Based Cause of Death Statistics Fail to Provide Reliable Data for Medical Aid in Dying. Int J Public Health (2023) 68:1606260. 10.3389/ijph.2023.1606260 37637487 PMC10450044

